# Dynamic models of immune responses: what is the ideal level of detail?

**DOI:** 10.1186/1742-4682-7-35

**Published:** 2010-08-20

**Authors:** Juilee Thakar, Mary Poss, Réka Albert, Gráinne H Long, Ranran Zhang

**Affiliations:** 1 Center for Infectious Disease Dynamics and Department of Physics, Pennsylvania State University, University Park, PA 16802, USA; 2 Penn State Hershey Cancer Institute, Pennsylvania State University, College of Medicine, Hershey, PA 17033 USA

## Abstract

**Background:**

One of the goals of computational immunology is to facilitate the study of infectious diseases. Dynamic modeling is a powerful tool to integrate empirical data from independent sources, make novel predictions, and to foresee the gaps in the current knowledge. Dynamic models constructed to study the interactions between pathogens and hosts' immune responses have revealed key regulatory processes in the infection.

**Optimum complexity and dynamic modeling:**

We discuss the usability of various deterministic dynamic modeling approaches to study the progression of infectious diseases. The complexity of these models is dependent on the number of components and the temporal resolution in the model. We comment on the specific use of simple and complex models in the study of the progression of infectious diseases.

**Conclusions:**

Models of sub-systems or simplified immune response can be used to hypothesize phenomena of host-pathogen interactions and to estimate rates and parameters. Nevertheless, to study the pathogenesis of an infection we need to develop models describing the dynamics of the immune components involved in the progression of the disease. Incorporation of the large number and variety of immune processes involved in pathogenesis requires tradeoffs in modeling.

## Background

Immune responses encompass a large range of temporal- (millisecond to days) and spatial (molecular to whole body) scales. It is increasingly recognized that intuitive arguments are not sufficient to make sense of this complexity. As an alternative, dynamic models are more and more frequently used to synthesize and complement empirical studies. Many dynamic models lead to valuable insights and predictions. For example, early dynamic models of infections provide a significant insight into the progression of AIDS [1,2].

The specific goal of a dynamic model of an infection may be to estimate certain parameters [3], to test competing hypotheses that can explain a set of observations [4,5] or to study the interplay between a pathogen and a host which can result in a progressive infection [6,7]. Immunological models consist of components representing immunological entities such as cells and cytokines, equations representing how the relationship between components changes their status, and parameters (e.g. rate constants) plugged into the equations which define the strength and timing of the relationships. Among the various mathematical frameworks employed by dynamic models (see Table 1), the deterministic (noise-free) framework is most frequently used at the cellular level. As the number of components included in a model increases, so does the number of parameters, and the value of most parameters tends to be unknown. Stereotypical models based on a simplified description that ignores the details of specific systems consist of few components, few kinetic rate constants and avoid the artifacts that might emerge from complex, parameter-rich models. Models of HIV infections developed upon the above principles have pioneered the field [1,2]. Nevertheless, models tracking a larger number of immune components are often desirable when studying the progression of an infection or disease.

**Table 1:** Overview of dynamic modeling methods

Dynamic modeling method	Granularity	Examples in immunology	Pros and cons	**Refs**.
Discrete dynamic models	Discrete time and discrete (abstract) state	Modeling of *Bordetella *infection pathogenesis, T cell receptor signaling	Can deal with many components but the simple state description cannot replicate continuous variation of immune components.	[6,44-47]

Continuous-discrete hybrid models (e.g. piecewise linear differential equations)	Combination of discrete and continuous state, continuous time	Modeling of infection pathogenesis and pathogen time-courses	The number of components that can be modeled is smaller than in discrete models because of the increase in the number of parameters. The state of the variables may not be directly comparable with experimental measurements. Although there are few parameters per component, parameter estimation becomes an issue for large systems.	[7,36]

Differential equations	Continuous time and state	SIR (Susceptible Infectious and Recovered) models of target cells and pathogens, T cell differentiation	The variables of the model can reproduce the experimentally observed concentrations. Insufficient data to inform the functional forms and parameter values can limit the use of this method. Less scalable than discrete approaches.	[11,13,20]

Finite state automata (e.g. agent-based models)	Discrete states (abstraction of cell state), discrete space and continuous time	Cell to cell communications	Simplified way to simulate spatial aspects. Can handle a few immune components in detail. Computationally expensive.	[48-50]

Partial differential equations	Continuous time, state and space	Transport of cells across vascular membranes	Appropriate to model a few immune components in detail. Computationally expensive and the determination of parameters is rather difficult.	[51,52]

Given our need to study the dynamics of immune responses to infection across different biological scales, and the limitations posed by the current state of empirical data, here we discuss the applications of simple versus complex models, and explore the use of discrete dynamic models. Excellent reviews of mathematical modeling in immunology [8] and of modeling multi-scale interactions [9,10] have already been published.

## Discussion

### Models of sub-systems or simplified immune response

Models can be kept relatively simple by detailing a few chosen processes and abstracting others. The number of components that need to be included in the model is reduced by focusing on a sub-system such as T cell expansion or the innate immune response, or by abstracting the immune response.

Dynamic models focusing on sub-systems of the immune response can be used to estimate specific parameters when appropriate empirical data is available. For example, mathematical models of T cell dynamics can be used to estimate T cell decay, production rates [11], killing rates [12], and the fate of recently produced T cells [13]. Such parameter estimates assist in the estimation of the *in vivo *basic reproduction number (R_0_) of viral infections. They are also useful for studying the efficacy of treatment for viral infections such as HIV [14,15]. Models revealing the differences in T cell dynamics of mice and humans [16] are critical in extending the empirical observations from mice to humans. Models tracking the dynamics of virus infection of host cells and cellular innate response, for example type I Interferon, predict the rates of target cell depletion in equine influenza virus infections [17].

Several dynamic models that simplify the immune response characterize the pathogen behavior in detail. Thus they can be used to determine the optimal conditions for within-host survival of a pathogen. For instance, the limited availability of red blood cells (resource limitation) can explain the early dynamics of malaria [4]. Similar models also reveal the pathogen-induced constraints leading to acute or persistent infections [18]. Although these models are based on assumptions such as correlation between virulence and growth rate of the pathogen [18,19], they give important insight into pathogenesis.

### Models of infection pathogenesis

The complexity of the models increases when they aim to capture multiple components of the immune response, which can include interactions between pathogen and host factors and the subsequent generation of specific antibody and T cell responses. The choice of mathematical description is critical in such instances due to the intricacies it can add or simplify. One example is a quantitative model constructed to simulate the immune response to infections by *Mycobacterium tuberculosis *(Mtb) [20,21] that tracks the dynamics of resident macrophages, immature dendritic cells, infected macrophages and mature dendritic cells. The dynamic causality in this model is approximated by mass-action and Michaelis-Menten kinetics. Since there are quantitative estimates available for Mtb (see table 4 in [20,21]), the model can parameterize the continuous change of immune components as a function of time. The model reveals specific parameters defining the dynamics of the host's immune processes that are important in persistent and acute infections. The simulated dynamics are validated by nonhuman primate data consisting of necropsies of Mtb infected animals [22].

In the absence of quantitative and mechanistic information, but having assembled a causal interaction network of the intra-cellular and cellular players elucidated by immunologists, a simpler qualitative/semi-qualitative formulation without or with only a few parameters can be followed. This discrete dynamic approach is supported by the observations that regulatory networks maintain their function even when faced with fluctuations in components and reaction rates [23-31]. Various discrete dynamic frameworks including Boolean networks [32], finite dynamical systems [33], difference equations [34], and Petri nets [35] have been used in modeling biological systems. Particularly, Boolean network models assume that each component has two qualitative states (e.g. active and inactive) and reproduce a sequence of switching events instead of modeling exact time courses. The active qualitative state can be interpreted as the concentration of an immune component that can induce downstream signaling. Such network models, tracking the dynamics of more than 30 immune components including various cytokines and cells, have been constructed for two *Bordetella *pathogens [6,7], for which few quantitative parameters have been determined. These models reproduce the qualitative features, such as the number of peaks, of the experimental time-courses of various immune components such as neutrophils and dominant cytokines.

Continuous-discrete hybrid models [7,36,37] are also developed with the aim to improve the representation of time while retaining the simplicity of switching functions. These hybrid models have a relatively small number of parameters, such as activation thresholds and decay rates, which are at a higher, more coarse-grained level than the kinetics of elementary reactions. A hybrid *Bordetella *model [7] reveals that many parameter combinations are compatible with the existing experimental knowledge on the pathogenesis. The distribution of the parameter values for each immune component in the model tells us about its role in the pathogenesis. Recent experimental measurements validate the IL4 time-course predicted by the model [Pathak, A. K., Creppage, K. E., Werner, J. R., Cattadori, I. M., "Immune regulation of a chronic bacterial infection and consequences for pathogen transmission", submitted].

Since the immune responses involve interactions at the site of infection, the maturation of T and B cells in the lymph nodes and the transport of cells through blood, capturing spatial dynamics may be critical for the success of a model. Approximations at various levels of detail are available that allow for the inclusion of some spatial information in the form of spatial compartments, coarse grids or reaction-diffusion processes. For example, the follow-up models of Mtb and *Bordetellae *[7,20] define two compartments, the site of infection (the lung) and the site of T cell differentiation (lymph node). A more detailed approach used by Gammack *et. al. *[38,39] describes granuloma formation in Mtb infections with a reaction-diffusion model using partial differential equations and the movement of innate immune cells toward a focal point of Mtb infection with a coarse-grid spatial formulation.

### Pros and cons of qualitative and quantitative approaches

The decision to use qualitative or quantitative models is based on the density of observations over time, the number of molecular or cellular players participating in a particular process and the connectivity of the regulatory network formed by these players. We note that both approaches necessitate knowledge of the causal or interaction network among components. Missing data and within-lab variations caused by the use of different experimental systems can introduce uncertainty in the determination of causal relationships; this issue is dealt with by the techniques of reverse engineering [40]. Observations taken at many time-points minimize the uncertainty about the behavior between the observations. The availability of frequent measurements for all or almost all the immune components one wants to model facilitates the use of quantitative modeling. The unavailability of such data guides us to use qualitative models which will inform us about the sequence of events and ultimate outcomes rather than trying to interpolate between the existing sparse observations. The assumption of switch-like regulatory relationships underlying qualitative models is a good approximation if the functional form of the regulatory relationship is sigmoidal.

Qualitative and quantitative approaches detail the immune interactions at different levels. Generally speaking, quantitative models give a detailed description of a relatively small number of interactions whereas qualitative models incorporate more interactions but have fewer kinetic details. Quantitative models offer predictions of kinetic parameters and of how the system will behave at a given instance. Qualitative models predict the response to knock-out or over-expression of components. An effective strategy to bridge these two approaches can be to iteratively refine qualitative models as more quantitative information becomes available through incorporation of more states, using a continuous-discrete hybrid formalism, or a fully quantitative description of an important sub-system.

Quantitative models require substantial prior knowledge and the interactions that require parameterization in these models have not yet been quantitatively characterized for most of the infections. The assumptions and estimations necessary to give values for the parameters may introduce unwanted artifacts in the model, reducing its usefulness. Since many molecular and cellular players of the immune cascades [41,42] are available for a range of infectious diseases, along with the outcomes of pathogen manipulation experiments, qualitative models can be constructed for less studied infectious diseases giving us insight about the dynamic interplay arising from the complex multi-scale interactions. Qualitative models also lose their simplicity and usefulness if the number of components and interactions included in the network is too large since that dramatically increases the system's dynamic repertoire. Various network simplification methods are available which reduce the number of components, for instance based on shortening long linear paths or collapsing alternative paths between a pair of nodes [43].

## Conclusion

The simple models developed to study parts of the immune system decipher parameters that reveal the regulation of immune responses and allow us to extrapolate the observations from experimental hosts to the natural hosts. The models developed to test the evolutionary fitness of pathogens reveal fundamental characteristics of the host-pathogen interactions and give useful insight into the pathogenesis of the infections. Among the models which aim to describe most of the immune components important in the pathogenesis, we show that both qualitative and quantitative models can be used effectively to study the progression of the infections.
